# Prevalence of psychological distress and associated factors among adult tuberculosis patients attending public health institutions in Dire Dawa and Harar cities, Eastern Ethiopia

**DOI:** 10.1186/s12889-019-7684-2

**Published:** 2019-10-28

**Authors:** Tegegn Mulatu Ayana, Kedir Teji Roba, Myrla Obejero Mabalhin

**Affiliations:** 1 Department of Nursing, Arba Minch University, College of Medicine and Health Sciences, Arba Minch, Ethiopia; 2 Haramaya University, College of Health and Medical Sciences, School of Nursing and Midwifery, Dire Dawa, Ethiopia

**Keywords:** Psychological distress, TB, Stigma, K-10 scale of psychological distress measurement

## Abstract

**Background:**

In developing countries, the prevalence of psychological distress was higher among tuberculosis patients. Patients with tuberculosis infection were more prone to psychological distress than peoples without tuberculosis. However, little studies were conducted on psychological distress among tuberculosis patients in Ethiopia, particularly in the Eastern Ethiopian health institutions.

**Methods:**

Institution-based cross-sectional study design was conducted. Based on the TB burden, four hospitals and six health centers were selected from Dire Dawa and Harar cities. Socio-demographic factors, psychological distress, TB related stigma experience, and alcohol use data were collected by face to face interview while TB and HIV related variables collected from TB registration book. All TB patients from the first month of TB treatment initiation through 6 were consecutively interviewed by trained data collectors from January to February 2018. The collected data were entered into Epi Data Version 3.1 software and exported into SPSS window version 20 for analysis. Bivariate and multivariate binary logistic regression was carried out. All variables with *P*-value ≤0.25 were taken into the multivariate model. Crude and adjusted odds ratios with a 95% confidence interval were estimated, and variables with *P*-value less than 0.05 in the final model were taken as significant predictors of psychological distress.

**Results:**

The prevalence of psychological distress among tuberculosis in this study population was 63.3% (95% CI: 58.1, 68.1). Being from rural residence (AOR: 1. 98; 95% CI: 1.01,3.86), co-infection TB- HIV (AOR: 2.15; 95% CI:1.02, 4.56), presence of at least one chronic disease (AOR:3.04; 95% CI:1.59,5.79), experience of stigma (AOR: 1.71; 95% CI:1.01, 2.90), Pulmonary and MDR-TB (AOR:2.53; 95% CI:1.50,4.28) and smoking cigarette (AOR:2.53; 95% CI:1.06,6.03) were associated with psychological distress.

**Conclusions:**

In this study, almost two-thirds of the tuberculosis patients had psychological distress. Chronic disease morbidity, HIV-TB co-infection and experienced TB related stigma were associated with psychological distress. Attention should be given to chronic diseases including HIV/AIDS diagnosis and referring to chronic disease units to prevent the impact on mental health. Consideration should be given for psychological distress and linking moderate to severe form of the disease to the Psychiatric clinics to hinder its effects.

## Background

Psychological distress (PD), is a general term that is used to describe an unpleasant subjective state of depression and anxiety which has both emotional and physiological manifestations that interfere with activities of daily living. It is characterized by symptoms of depression and anxiety and may also somatic symptoms. In normal functioning individuals, psychological distress is the fluctuation of mood. Psychological distress can result in negative views of the environment, self, and others [1].

A state of distress can be caused by many things such as poverty, unemployment, death of a loved one, a relationship break-up, medical illness or physical problems, alcohol and drug use [2, 3]. As a consequence of illness, many patients face psychological challenges [4]. Tuberculosis (TB) is one of those illnesses that result in PD. Similarly, lack of support by the family and community or TB related stigma were the risk factors for PD [5]. Psychological distress and physical health were the factors that affect the quality of life of the patients [6].

The severity of PD is dependent upon the situation and how someone perceives it. No two people experience one event the exact same way [1, 7]. Just as mental illness can impact on areas of the individual’s life, psychological distress can also have direct and indirect effects on the individual’s psychological, social and occupational functioning, affecting many areas of their life, including relationships, work and health [8].

Many people who suffer significant psychological distress do not come into contact with specialized mental health services. While many of these people may seek help from general practitioners, counselors and support groups, significant numbers do not access any type of formal help in the face of psychological distress [8]. In 2015, more than 300 million peoples were estimated to suffer from depression, which accounts for 4.4% of the world’s population. Worldwide the number of peoples with depression and anxiety is increasing especially in low-income countries. More than 80% of this disease burden occurred in low and middle-income countries. Nearly half (48%) of the world’s depressed population were living in South East Asia and Western Pacific regions. The prevalence of depression in the Eastern Mediterranean, Americas, European, and African region were 16, 15, 12, and 9% respectively. In Ethiopia, 4.7% of the total population was suffering from a depressive disorder in the general population [2].

In Europe, 44.4% of tuberculosis (TB) patients had suffered from psychological distress. In the region magnitude of depression among TB patients ranged from 49.4 to 60.5% while anxiety ranged from 26.0 to 38.3% [9, 10]. In the Eastern Mediterranean, 46.3% of TB patients were suffering from depressive disorder [11]. In Western Pacific Regions, 16.8 to 65.2% of TB patients suffered from psychological distress [12, 13]. In low and middle-income countries depressive episodes among TB patients were more than three times higher than peoples without tuberculosis [14].

Few studies in Africa indicated more than half of the patients were suffering from psychological distress among TB patients. Two studies from Africa indicated that the prevalence of PD among TB patients was 67.6 and 81.1% [15, 16]. The lowest ranged from 8 to 25.4% [17, 18]. While other studies ranged from 40 to 81.1% [15, 16, 19, 20]. The prevalence of depression ranges from 43.4 to 61.1% [20–22].

One study from the capital city of Ethiopia indicated that 67.6% of TB patients suffer from PD. The Country nationwide study showed that the magnitude of depression among the general population was found at 9.1% [23]. One study from the country indicates that 19.82% of TB patients had PD [24]. In other studies, it ranged from 40.6 to 67.6% [19, 20]. History of past TB treatment was the risk factor for PD. The majority (87.7%) of past history of TB treatment were found psychologically distressed [16].

Substances like Khat, tobacco, and alcohol use are the most common in the Eastern parts of Ethiopia. The prevalence of chewing Khat in Harar, Eastern Ethiopia was ranged from 48.2–53.2% [25, 26] while tobacco and alcohol uses were 38.2 and 10.5% respectively [26]. Substances use increased the risk of depression and anxiety disorder [27].

Both Tuberculosis and PD share common risk factors, as a result, both of them are a co-morbid disease [14, 28]. Depression is a major risk factor for poor treatment outcome and death. In addition to these depression causes poor quality of life and greater disability throughout TB treatment period [16, 29]. Being diagnosis with TB increased the risk for common mental disorders [30].

Factors associated with Psychological distress in TB were included: Older age, unmarried, grade 8–11 and grade 12 or more educational level [15], low economic status, TB/HIV co-infected [15, 16] history of previously TB treatment, on ART treatment follow-up, and alcohol use [16], perceived her/his illness as a moderate or severe [13], perceived TB stigma [13, 20, 31], co-morbid chronic illness [20], alcohol use [16], current smoking [14].

To the knowledge of these researchers, there are limited studies that assessed the magnitude of psychological distress among TB patients in Ethiopia. Therefore, this study will determine the magnitude of psychological distress and associated factors in Eastern Ethiopia.

## Methods

### Study design

Cross-sectional study design was conducted from January to February 2018 among TB patients in Harar and Dire Dawa cities health institutions, Eastern Ethiopia.

### Sample size, sampling, and procedure

The sample size determined based on the formula for a single population proportion by taking a proportion of psychological distress of 67.6% [16], by assuming a 95% confident interval of Z α/_2_ = 1.96, a margin of error 5%, adding 10% non-response rate final sample size was 370.

Harar and Dire Dawa, which is located in the Eastern part of Ethiopia, 526 and 515 km from Addis Ababa, the capital city of Ethiopia respectively. The cities are 48 km apart and share similar socio-demographic and cultural characteristics and have their own administrative regions. In both regions, there are 4 Hospitals and 24 Health Centers in the Urban and Rural areas. The city’s health institution has the highest TB disease burden. Based on the TB burden per hospital, four Hospitals from both cities were selected. Similarly, from 24 health centers, six of them were selected from both cities that have TB disease burden. All TB patients from the first month of TB treatment initiation through 6 were consecutively interviewed from January to February 2018. The interview was conducted by trained 10 diploma nurses and data collection was supervised by 3 BSc nurses. Data collectors were obtained signed informed consent from study participants preceding data collection.

### Measurements

A structured questionnaire was developed by reviewing research literatures. The questionnaire has different parts. The socio-demographic characteristics (age, sex, residence, marital status, occupation, and the others), history of chronic diseases, self-rated illness severity, experienced TB stigma, psychological distress, and substance use data were collected by face to face interview while others variables like HIV status, ART treatment, types of TB disease, and phases of TB treatment were collected from TB registration books.

*The Kessler Psychological Distress Scale (K-10*) questionnaire was used to assess psychological distress. K-10 item scale contains 10 questions. It serves as to identify non-specific anxiety and depressive symptoms that a person has experienced in the most recent 30 days’ period. The frequency of each item on the K-10 scale experienced by a patient was recorded using a five-point Likert scale. Each scale range from 1 to 5 (none of the time, little of the time, some of the time, most of the time and all of the time). The item score sum ranges from a minimum of 10 to a maximum 50 which indicates increasing the degree of psychological distress. A cut-off score greater than or equal to 16 was considered as having psychological distress [13, 15, 16, 31].

#### Experienced TB stigma

an individual who experiences treatment by family members, neighbors, and friends in daily life since the diagnosis of TB. The 9 item stigma questionnaire was used. The items have four points Likert scale ranging from 1 to 4 (strongly disagree/ disagree/ agree/ strongly agree) respectively. The total scores ranged from 9 to 36. Stigma was defined if the summed score was more than the median score [13]. The Cronbach’s alpha for the stigma in this study was good (Cronbach’s alpha = 0.93).

#### Self-rated illness severity

Illness severity degree that makes the respondent worry and results in distress was asked: How do rate the severity of your illness? Mild type, Moderate type or Severe type. The measurement was based on participants’ feelings response.

#### Alcohol consumption

Alcohol Use Disorders Identification Test (AUDIT) tool was used to assess alcohol consumption. No significant differences were observed between AUDIT-3, AUDIT-4, and AUDIT-10 items in assessing risky alcohol consumption. Because of this AUDIT-3 was used to assess alcohol consumption. An item contains three questions “How often do you have a drink containing alcohol?” Response ranges from 0 to 4 (Never, monthly or less, 2 to 4 times a month, 2 to 3 times a week and 4 or more times a week), “How many drinks containing alcohol do you have on a typical day when you are drinking?” Response ranges from 0 to 4 (1 or 2, 3 or 4, 5 or 6, 7–9 and 10 or more), and “How often do you have 6 or more drinks on one occasion?” Response ranges from 0 to 4 (Never, less than monthly, monthly, weekly, and daily or almost daily). AUDIT-3 scores ranged from 0 to 12 with a score of 3 and above considered risky drink [32].

### Data processing and analysis

The collected data were checked for completeness and consistency, coded and entered into Epi Data Version 3.1 software and exported into Statistical Package for the Social Sciences (SPSS window version 20) for analysis. Data were explored and cleaned prior to analysis using SPSS. The frequency, means, and standard deviations were employed to describe the samples. Bivariate and multivariate binary logistic regression analyses were carried out to determine the predictors of psychological distress. Variables with a *P*-value of less than 0.25 in the bivariate binary logistic regression analysis were considered for multivariable regression model. Crude and adjusted odds ratio with a 95% confidence interval was estimated, and variables with *P*-value less than 0.05 in the multivariable regression analysis were taken as significant predictors of psychological distress.

### Ethical consideration

Ethical approval was obtained from the Ethical Review Board of Haramaya University, College of Health and Medical Sciences. Written informed consent was obtained from each study participant. The interview was conducted in a private area. No personal identifiers were recorded to ensure confidentiality. The participants were the right not answer to questions what comfortable to it. Patients who scored 30 and above considered as severe psychological distress with Kessler 10 item questions were linked to Psychiatric Clinic.

## Results

### Socio-demographic characteristics

A total of 365 participants were interviewed which gives a response rate of 98.6%. The mean age of the study participants was 35.52 years (±13.93 SD) in age ranges from 18 to 85 years old and nearly less than half (45.8%) were aged below 30 years old. More than half (56.2%) of study participants were males and 78.1% were urban residents. More than half (55.9%) were married and one-third of the respondents were single 117 (32.1%). Two-third of the respondents (58.6% were grade 7 or less educational status. Concerning occupational status 68 (18.6%) were government employed while 13 (3.6%) NGO employed. Two hundred and twenty-eight (62.5%) of respondents have income ≥1000 Ethiopian Birr (ETB) and 18.1% of the study participants have no income and live with support from family or others (Table 1).

**Table 1:** Socio-demographic characteristics of the study population in Dire Dawa and Harar cities Health Institutions, Eastern Ethiopia, 2018 (*n* = 365)

Variable	Frequency	Percent (%)
Age (years)		
18–30	167	45.8
31–44	121	33.2
45 or above	77	21.1
Sex		
Male	205	56.2
Female	160	43.8
Residence		
Urban	285	78.1
Rural	80	21.9
Marital status		
Single	117	32.1
Married	204	55.9
Divorced	26	7.1
Widowed	18	4.9
Educational status		
Grade 7 or less	214	58.6
Grade 8–11	80	21.9
Grade 12 or above	71	19.5
Occupational status		
Farmer	56	15.3
House wife	59	16.2
Merchant	62	17.0
Government employee	69	18.9
NGO	13	3.6
Student	48	13.2
Daily Labor	22	6.0
Others	36	9.9
Monthly respondent’s income (ETB)		
no income	66	18.1
< = 500	25	6.8
501–999	46	12.6
> = 1000	228	62.5

Ethiopian Birr

### Health-related and substance use characteristics

Less than two-third (60.3%) of study participants were diagnosed with pulmonary TB while 18 (4.9%) and 127 (34.8%) MDR and extra-pulmonary TB respectively. The majority (89.6%) of study participants were new TB treatment category. Almost two-third (64.4%) of TB patients were in the continuous treatment phase. Concerning the HIV status of the study participants, one fifth (18.1%) of study participants were reactive for HIV tests. Less than one third (29%) of study participants had one or more chronic diseases. Almost half (46%) of the study participants had experienced TB related stigma. Few (5.5%) of the study participants had a family history of mental illness. Less than half (44.4%) of the study participants have rated their TB illness as a moderate type while 132 (36.2%) rated as a severe form (Table 2).

**Table 2:** Health-related and substance use characteristics of the study population in Dire Dawa and Harar cities Health Institutions, Eastern Ethiopia, 2018 (*n* = 365)

Variable	Frequency	Percent (%)
TB category		
Pulmonary	220	60.3
MDR TB	18	4.9
Extra Pulmonary	127	34.8
Treatment category		
New	327	89.6
Repeated	38	10.4
TB treatment Phase		
Intensive Phase	130	35.6
Continuous Phase	235	64.4
HIV status		
Non- Reactive	299	81.9
Reactive	66	18.1
Dual treatment for TB & HIV		
No	299	81.9
Yes	66	18.1
Having one or more chronic Disease		
No	259	71.0
Yes	106	29.0
Self-rated illness severity		
Mild	71	19.5
Moderate	162	44.4
Severe	132	36.2
Experienced TB stigma		
Not experienced	197	54.0
experienced	168	46.0
Family History of mental illness		
No	345	94.5
Yes	20	5.5
Alcohol drinking risk		
Low	349	95.6
High	16	4.4
Current khat chew		
No	177	48.5
Yes	188	51.5
Amount of khat use		
Never use	177	48.5
One’s Monthly	21	5.8
One’s weekly	65	17.8
2 or more weekly	76	20.8
Almost daily	26	7.1
Current cigarate smoker		
No	306	83.8
Yes	59	16.2
Amount of cigarette use		
Never use	306	83.8
One’s Monthly	4	1.1
One’s weekly	16	4.4
2 or more weekly	13	3.6
Almost daily	26	7.1

### Prevalence of psychological distress

Among 365 Tuberculosis patients, 231 (63.3%) had psychological distress (95% CI: 58.1, 68.1). The mean of psychological distress was 19.23 (± 7.18 SD) ranging from 10 to 50. Among the study participants’ 25.5, 31.8 and 6% showed a mild, moderate and severe form of psychological distress respectively. Psychological distress among female gender 106 (66.2%), greater than 45 years old 54 (70.1%), rural residence 67 (78.8%), TB and HIV co-infected 54 (81.8%), repeated TB treatment 29 (76.3%), one or more chronic diseases 87 (82.1%), experienced TB stigma 130 (76.9%), alcohol risk drinkers 14 (87.5%), cigarette smokers 50 (83.3%) had higher proportion of psychological distress. Psychological distress among pulmonary TB, MDR-TB and extra-pulmonary TB were 73.2, 72.2 and 44.8% respectively (Table 3).

**Table 3:** Psychological Distress among TB patients in Dire Dawa and Harar cities Health Institutions, Eastern Ethiopia, 2018 (*N* = 365)

Variables	Psychological Distress, n (%)		N (%) in the groups
	Yes	No	
Age			
18–30	101 (60.5)	66 (39.5)	167 (45.8)
31–44	76 (62.8)	45 (37.2)	121 (33.2)
≥ 45	54 (70.1)	23 (29.9)	77 (21.1)
Sex			
Male	125 (60)	80 (40)	205 (56.2)
Female	106 (66.2)	54 (33.8)	160 (43.8)
Residence			
Rural	67 (78.8)	18 (21.2)	164 (71)
Urban	164 (58.6)	116 (41.4)	67 (29)
Marital status			
Single/Divorced/widowed	112 (69.6)	49 (30.4)	161 (44.1)
Married	119 (58.3)	85 (41.7)	204 (55.9)
TB-HIV Co-infection			
Yes	54 (81.8)	12 (18.2)	66 (18.1)
No	177 (59.2)	122 (40.8)	299 (81.9)
TB treatment			
Repeated	29 (76.3)	9 (23.7)	38 (10.4)
New	202 (61.8)	125 (38.2)	327 (89.6)
TB type			
Pulmonary & MDR TB	174 (73.1)	64 (26.9)	238 (65.2)
Extra Pulmonary	57 (44.9)	70 (55.1)	127 (34.8)
One or more chronic Disease			
Yes	87 (82.1)	19 (17.9)	106 (29)
No	144 (55.6)	115 (44.4)	259 (71)
Experienced TB stigma			
Yes	130 (76.9)	39 (23.1)	169 (46.3)
No	101 (51.5)	95 (48.5)	196 (53.7)
Self-rated illness severity			
Mild	35 (49.3)	36 (50.7)	71 (19.5)
Moderate	105 (64.8)	57 (35.2)	162 (44.4)
Severe	91 (68.9)	41 (31.1)	132 (36.2)
Alcohol use risk			
Risk drinking	14 (87.5)	2 (12.5)	16 (4.4)
No risk	217 (62.2)	132 (37.8)	349 (95.6)
Current khat Chewing			
Yes	128 (68.1)	60 (31.9)	188 (51.5)
No	103 (58.2)	74 (41.8)	177 (48.5)
Current Cigarette smoker			
Yes	50 (83.3)	10 (16.7)	60 (16.4)
No	181 (59.3)	124 (40.7)	305 (83.6)
Income			
No income	41 (62.1)	25 (37.9)	66 (18.1)
< = 500 ETB	19 (76)	6 (24)	25 (6.8)
501–999 ETB	35 (76.1)	11 (23.9)	46 (12.6)
> = 1000 ETB	136 (59.6)	92 (40.4)	228 (62.5)

### Predictors of psychological distress

Bivariate logistic regression analysis revealed that age, residence, marital status, current khat chewing, current cigarette smoking, self-rated illness severity, TB treatment phase, TB re-treatment, HIV status, alcohol use, one or more chronic diseases, experienced TB stigma, and TB-HIV co-infection were associated with psychological distress. In the model; sex, educational status, TB treatment phase, and family history of mental illness were not associated in the bivariate logistic regression model. In multivariate logistic regression analysis showed that single/divorced/widowed, rural residence, TB and HIV co-infection, one or more chronic diseases, experienced TB stigma, being pulmonary and MDR tuberculosis patient and current cigarette smoking were significantly associated with psychological distress (Table 4).

**Table 4:** Factors associated with Psychological Distress among adult TB patients in Harar and Dire Dawa cities Health institutions, Eastern Ethiopia, 2018 (n = 365)

Variables	Psychological Distress, n (%)		COR (95% CI)	AOR (95% CI)
	Yes	No		
Age				
18–30	101 (60.5)	66 (39.5)	1	1.00
31–44	76 (62.8)	45 (37.2)	1.10 (0.68,1.79)	0.82 (0.43,1.56)
≥ 45	54 (70.1)	23 (29.9)	1.53 (0.860,2.74)	0.85 (0.40,1.80)
Residence				
Rural	67 (78.8)	18 (21.2)	2.63 (1.49,4.67)**	1. 98 (1.01,3.86)*
Urban	164 (58.6)	116 (41.4)	1	1
Marital status				
Single/Divorced/widowed	112 (69.6)	49 (30.4)	1.63 (1.10,2.53)*	1.88 (1.06,3.35)*
Married	119 (58.3)	85 (41.7)	1	1
TB and HIV Co-infection				
Yes	54 (81.8)	12 (18.2)	3.10 (1.60,6.04)***	2.15 (1.02,4.56)*
No	177 (59.2)	122 (40.8)	1	1
TB treatment type				
Repeated	29 (76.3)	9 (23.7)	1.99 (0.91,4.35)	1.34 (0.54,3.31)
New	202 (61.8)	125 (38.2)	1	1
TB type				
Pulmonary & MDR TB	174 (73.1)	64 (26.9)	3.34 (2.13,5.25)***	2.53 (1.50,4.28)***
Extra Pulmonary	57 (44.9)	70 (55.1)	1	1
One or more chronic Disease				
Yes	87 (82.1)	19 (17.9)	3.66 (2.10,6.36)***	3.04 (1.59,5.79)***
No	144 (55.6)	115 (44.4)	1	1
Experienced TB stigma				
Yes	130 (76.9)	39 (23.1)	3.14 (1.99,4.94)***	1.71 (1.01,2.90)*
No	101 (51.5)	95 (48.5)	1	1
Self-rated illness severity				
Mild	35 (49.3)	36 (50.7)	1	1
Moderate	105 (64.8)	57 (35.2)	1.90 (1.08,3.34)*	1.42 (0.72,2.81)
Severe	91 (68.9)	41 (31.1)	2.28 (1.26,4.13)***	1.35 (0.66,2.77)
Alcohol use disorder risk				
Risk	14 (87.5)	2 (12.5)	4.26 (0.95,19.03)	4.41 (0.85,23.05)
No risk	217 (62.2)	132 (37.8)	1	1
Current Chat Chewing				
Yes	128 (68.1)	60 (31.9)	1.53 (1.00,2.35)*	0.96 (0.55,1.69)
No	103 (58.2)	74 (41.8)	1	1
Current cigarette or shisha				
Smoker	50 (83.3)	10 (16.7)	3.43 (1.67,7.01)***	2.53 (1.06,6.03)*
Non-Smoker	181 (59.3)	124 (40.7)	1	1
Income				
No income	41 (62.1)	25 (37.9)	1.12 (0.63,1.95)	1.25 (0.61,2.57)
< = 500 ETB	19 (76)	6 (24)	2.14, (0.82,5.57)	1.51 (0.48,4.75)
5011–999 ETB	35 (76.1)	11 (23.9)	2.15 (1.04,4.45)*	1.48 (0.65,3.38)
> = 1000 ETB	136 (59.6)	92 (40.4)	1	1

*** *P*- value < 0.001, ** *P*-value < 0.01, * *P*-value < 0.05

In this study, patients whose lives in a rural area were two times higher odds of psychological distress as compared to urban residence (AOR: 1.98, 95% CI: 1.01, 3.86). Marital status was a factor associated with psychological distress. Patients who were single/ divorced/widowed were 1.9 times higher odds of psychological distress as compared to those who were married (AOR: 1.88, 95% CI: 1.06, 3.35). Concerning TB- HIV co-infection, co-infected patients were a risk of psychological distress. Tuberculosis and HIV co-infected patients were 2.2 times higher odds of psychological distress as compared to those not co-infected patients (AOR: 2.15, 95% CI: 1.02, 4.56). Similarly, patients who had one or more chronic diseases were 3 times higher odds of psychological distress as compared to patients who have no chronic disease (AOR: 3.04, 95% CI: 1.59, 5.79). Tuberculosis disease type was associated with psychological distress. Pulmonary and MDR TB patients were 2.5 times more likely associated with psychological distress as compared to Extra Pulmonary TB (AOR: 2.53, 95% CI: 1.50,4.28). Regarding experienced TB stigma, patients who had experienced TB stigma were 1.7 times higher odds of psychological distress as compared to counterpart (AOR: 1.71, 95% CI: 1.01, 2.90). Patients who smoke cigarettes currently were 2.5 times more likely associated with psychological distress as compared to non-smokers (AOR: 2.53, 95% CI: 1.06,6.03) (Table 4).

## Discussion

The prevalence of psychological distress among TB patients in this study was 63.3% (95% CI: 58.1, 68.1). The prevalence of psychological distress among TB patients in this study is similar to studies conducted in China (65.2%), Addis Ababa, Ethiopia (67.6%), and three Oromia State hospitals, Ethiopia (64%) [13, 16, 31]. This finding is lower as compared to studies in South Africa (81.1%) [15]. The possible explanation might be a small sample size, study population includes TB diagnosis to 6 months of treatment periods, the population being studied. Perceived TB stigma might be reduced over treatment periods [31]. Common mental disorders decrease over treatment periods [16]. It is higher than study in Enugu, Nigeria (25.4%), in Wolaita Sodo University Hospital and Sodo Health Center, South state of Ethiopia (40.6%), in Jimma University Specialized Hospital and Jimma Health Center, Ethiopia (19.8%), and in Addis Ababa thirty one health facilities, Ethiopia (48.9%) [18, 20, 24, 33]. These differences could be due to the population being studied, measurement tools, data collection periods [31].

Married or cohabitated individuals have lower risk factors for psychological distress. In this study married or cohabited individuals were at a lower risk of developing PD as compared combined marital status. This study finding is consistent with a study done in Addis Ababa, Ethiopia and South Africa [16, 19], which indicated that cohabitated were less likely associated with psychological distress. Support by family, friends or community is lower the risk of stress and depression [34].

Tuberculosis HIV co-infection is one of the risk factors for the development of PD. In this study TB HIV co-infected was a higher risk for the development of PD than TB infected. This finding is consistent with studies done in South Africa, Wolaita Sodo, South Ethiopia, Cameroon, and three hospitals in the Oromia regional state of Ethiopia [15, 20, 22, 31]. This might be due to depression is the most common mental disorder among HIV infected patients. Similarly, the risk of depression doubled among HIV infected patients [35, 36].

The likelihood of developing a depressive disorder is higher among chronic medical illnesses. In this study, chronic medical illnesses were associated with PD. This finding is consistent with the study done in South Africa [37]. Depression disorder has been increased two to three times higher among chronic medical illnesses [38]. On the contrary, chronic medical illness masks the probability of diagnosing and treating a depression disorder [39].

Stigma experience due to TB is one of the risk factors for common mental disorders. In this study stigmatization due to TB by the family, friends, or community was associated with PD. This finding is in line with the study done in Shandong Province, Eastern China, Wolaita Sodo, South Ethiopia, and three hospitals in the Oromia regional state of Ethiopia [13, 20, 31]. This might be due to being diagnosed with TB result in psychological problems [40]. In addition to this poor general health perception due to TB is the risk factor for the common mental disorders [31].

In this study substances like cigarette smoking is riskier for the development of psychological problems. This study finding congruent with the study done in 48 low-and middle-income countries showing that TB patients who smoke cigarettes were associated with a higher depression as compared to non – smokers [14]. This might be because of depression is more common among substance users [41].

### Limitation of the study

Variables like psychological distress and stigma assessed by interviewer-administered as a result, recall bias and desirable responses may have been given. Since the study was cross-sectional does not show a cause-effect relationship. The results of this study may not be generalized to the community or TB patients who lived in the community because of institution-based study.

## Conclusions

In this study, almost two-thirds of the tuberculosis patients had psychological distress. Chronic disease morbidity, HIV-TB co-infection and experienced TB related stigma were associated with psychological distress. Attention should be given to chronic diseases including HIV/AIDS diagnosis and referring to chronic disease units to prevent the impact on mental health. Consideration should be given for psychological distress and linking moderate to severe form of the disease to the Psychiatric clinics to hinder its effects.

## Data Availability

The datasets used and analyzed for this work are available from the corresponding author on reasonable request.

## Abbreviations

ART: Antiretroviral Therapy
HIV: Human immunodeficiency virus
MDR-TB: Multi-Drug Resistant Tuberculosis
PD: Psychological Distress
TB: Tuberculosis
